# Review on Abyssomicins: Inhibitors of the Chorismate Pathway and Folate Biosynthesis

**DOI:** 10.3390/molecules23061371

**Published:** 2018-06-06

**Authors:** Carmen Sadaka, Edmund Ellsworth, Paul Robert Hansen, Richard Ewin, Peter Damborg, Jeffrey L. Watts

**Affiliations:** 1 Department of Veterinary and Animal Sciences, University of Copenhagen, Stigboejlen 41870, Frederiksberg C, Denmark; pedam@sund.ku.dk; 2 Department of Pharmacology and Toxicology, Michigan State University, 220 Trowbridge Road, East Lansing, MI 48824, USA; ellswo59@msu.edu; 3 Department of Drug Design and Pharmacology, University of Copenhagen, Universitetsparken 2100, Copenhagen, Denmark; prh@sund.ku.dk; 4 Zoetis Global Therapeutics Research, 333 Portage Street, Kalamazoo, MI 49007, USA; richard.ewin@zoetis.com

**Keywords:** antifolate, para-aminobenzoic acid, chorismate, resistance, spirotetronate, antibiotic, sulfonamides, prodrug, analoging

## Abstract

Antifolates targeting folate biosynthesis within the shikimate-chorismate-folate metabolic pathway are ideal and selective antimicrobials, since higher eukaryotes lack this pathway and rely on an exogenous source of folate. Resistance to the available antifolates, inhibiting the folate pathway, underlines the need for novel antibiotic scaffolds and molecular targets. While para-aminobenzoic acid synthesis within the chorismate pathway constitutes a novel molecular target for antifolates, abyssomicins are its first known natural inhibitors. This review describes the abyssomicin family, a novel spirotetronate polyketide Class I antimicrobial. It summarizes synthetic and biological studies, structural, biosynthetic, and biological properties of the abyssomicin family members. This paper aims to explain their molecular target, mechanism of action, structure–activity relationship, and to explore their biological and pharmacological potential. Thirty-two natural abyssomicins and numerous synthetic analogues have been reported. The biological activity of abyssomicins includes their antimicrobial activity against Gram-positive bacteria and mycobacteria, antitumor properties, latent human immunodeficiency virus (HIV) reactivator, anti-HIV and HIV replication inducer properties. Their antimalarial properties have not been explored yet. Future analoging programs using the structure–activity relationship data and synthetic approaches may provide a novel abyssomicin structure that is active and devoid of cytotoxicity. Abyssomicin J and atrop-*o*-benzyl-desmethylabyssomicin C constitute promising candidates for such programs.

## 1. Introduction

**Figure 1:**
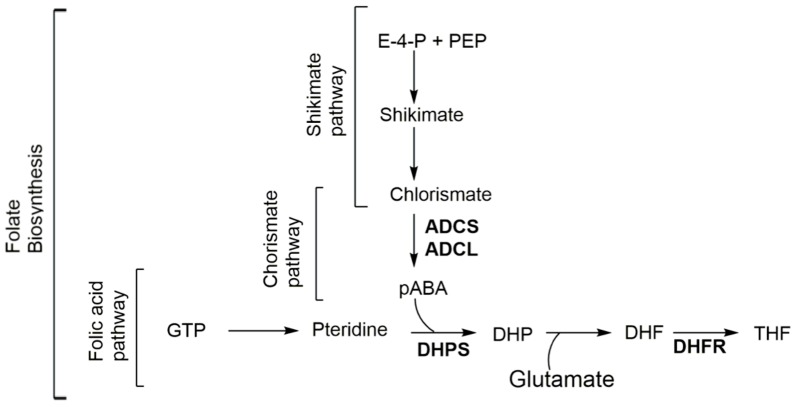
Folate biosynthesis through the shikimate-chorismate-folate (SCF) biosynthetic pathway. ADCL amino-deoxychorismate lyase; ADCS amino-deoxychorismate synthase; DHF dihydrofolate; DHP dihydropteroate; DHFR dihydrofolate reductase; DHPS dihydropteroate synthase; E-4-P erythrose-4-phosphate; GTP guanosine-5′-triphosphate; pABA para-aminobenzoic acid; PEP phosphoenolpyruvate; and THF tetrahydrofolate.

Folates are cofactors in one-carbon transfer reactions involved in several other pathways, such as the synthesis of nucleotides, the methylation cycle, and cell division in prokaryotic and eukaryotic organisms [1,2,3,4,5,6]. Plants, prokaryotes, and some lower eukaryotes rely on de novo synthesis of folate via the shikimate–chorismate–folate (SCF) biosynthetic pathway (Figure 1) [2,3,7]. Higher eukaryotes lack this biosynthetic pathway and rely on an exogenous source of folate [8]. Inhibiting the biosynthesis of this vital metabolite blocks cell division and leads to cell death [1,2,3,4,5,6]. Within the SCF metabolic pathway, the folic acid [9,10], chorismate [9], and shikimate [11,12] branches are considered primary, secondary, and ternary metabolisms, respectively, for the biosynthesis of folates (Figure 1). The relationship of these three pathways (termed SCF for this review) is shown in Figure 1. In summary, within the folic acid pathway, folates are enzymatically synthesized from guanosine triphosphate (GTP) by a series of seven enzymes requiring the condensation of para-aminobenzoic acid (pABA) to the pterin moiety [1] (note S1, supplemental data). pABA, itself, is synthesized through conversion of precursors within the shikimate (note S2, supplemental data) and chorismate (note S2, supplemental data) pathways. The central biological importance of folate and the absence of the SCF biosynthetic pathway in mammals make it an attractive target for the development of antimicrobials, antiparasitics, and herbicides [1,13,14,15,16,17]. The success of the herbicide glyphosate (targeting the shikimate pathway) [18,19,20], and that of sulfonamides and potentiated sulfonamide antibiotics (targeting the folic acid pathway) validate this approach [21,22]. Of the known clinically useful antimicrobials and antimalarials, only sulfonamides (note S4, supplemental data) and diaminopyrimidines (note S5, supplemental data), often used in combination, target the folic acid pathway within the SCF metabolic pathway [2,7]. One of the effective resistance mechanisms to these antifolates consists of an overproduction of pABA within the chorismate pathway [2,3,7,23,24,25]. With the emergence of resistance to these antifolates reducing their therapeutic utility [2,7], there is an urgent and compelling need for the development of novel antifolates that are effective against both insensitive and resistant strains [2,7]. Unlike the folate and shikimate pathways that have been heavily exploited by the pharmaceutical [10,21,22,23,26] and herbicide industries [10,11,12,13,14,15,16,17,18,19,20], respectively, the chorismate pathway offers considerable promise as a potential target for new antimicrobials.

The emerging interest around the abyssomicin pharmacophore has been driven by the observations that abyssomicin C, isolated from *Verrucosispora* sp. (AB-18-032) in 2004 [27,28,29], inhibits the biosynthesis of pABA, a key cofactor required for folic acid biosynthesis, by trapping irreversibly the 4-amino-4-deoxychorismate synthase (ADCS) enzyme within the chorismate pathway through a Michael addition to a cysteine residue [30,31,32,33]. Abyssomicin C exhibits promising effects against methicillin resistant *Staphylococcus aureus* (MRSA) [34] and mycobacteria causing tuberculosis [27,35,36], validating pABA synthesis as a potential useful antifolate target. This discovery highlights the abyssomicin pharmacophore as the next generation of antifolates, and the first generation of pABA synthesis inhibitors. Some abyssomicins also have antitumor activity [37] and can reactivate latent human immunodeficiency virus (HIV) [38]. The antibacterial activity of abyssomicins has been explored through biosynthetic evaluation, total synthesis, and pharmacological studies. Elegant synthetic routes have given access to new chemistries and the synthesis of several naturally occurring abyssomicins, as well as various novel analogues [12,26,31,32,33,34,39,40,41,42,43,44,45]. 

This review discusses the novel antibiotic scaffold of abyssomicins. We summarize structural, biosynthetic, and biological properties of the abyssomicin family members along with synthetic, biological, and pharmacological studies conducted. This report aims to elucidate their molecular target, mode of action, as well as key structure–activity relationship (SAR) requirements of the abyssomicin pharmacophore, and to explore their different biological and pharmacological potential.

## 2. The First Discovery: Abyssomicins B–D

**Figure 2:**
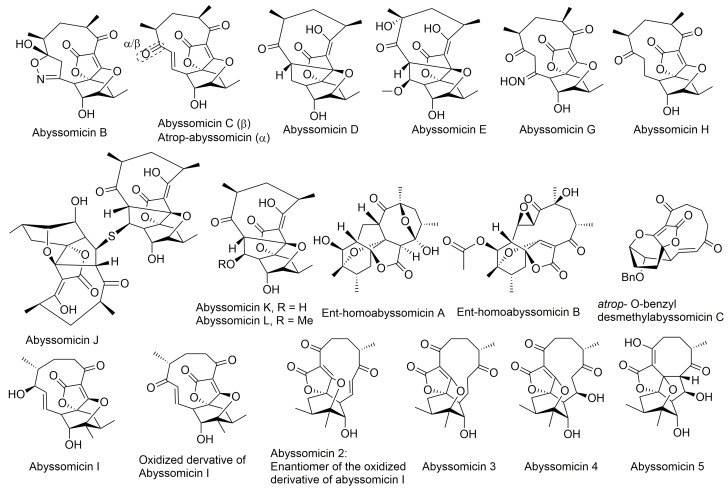

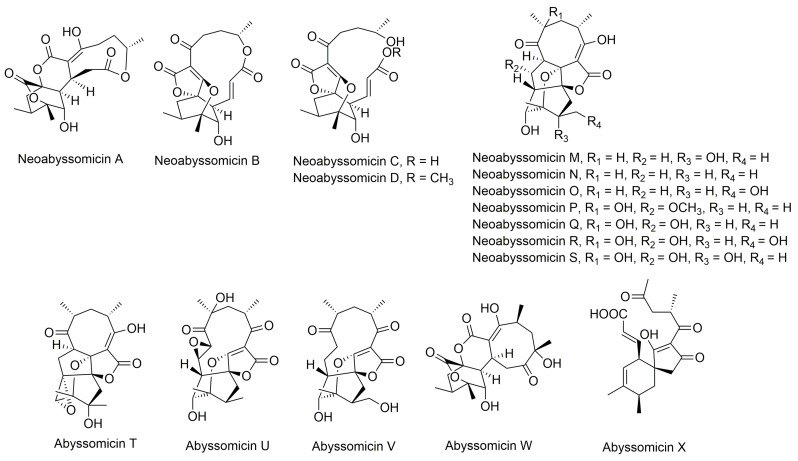
Structures of abyssomicins B–E, G–L, M–X, 2–5, atrop-abyssomicin C, ent-homoabyssomicin A and B, atrop-*o*-benzyl-desmethylabyssomicin C, the oxidized derivative of abyssomicin I, and neoabyssomicins A–D (modified from [35,37,38,42,49,50,51,52]). Abyssomicin 2 is the enantiomer of the oxidized derivative of abyssomicin I. Abyssomicin X is the first reported naturally occurring linear abyssomicin.

Natural product screening has long played a key role in the discovery of novel antibacterials, with a large fraction of those natural bioactive extracts isolated from actinomycetes [46]. Similarly, three novel natural compounds dubbed as abyssomicins B, C, and D (Figure 2), were purified and characterized in 2004 [27,28], from the marine actinomycete *Verrucosispora* strain AB-18-032, known today as the new taxon *Verrucosispora maris* sp. nov. [27,28,47]. Abyssomicin C was the sole active member among the three purified abyssomicins B–D [27,28,29]. It showed an inhibitory activity against MRSA N315 and MRSA Mu50, which could be depleted upon addition of pABA [28,47]. This demonstrated that its activity targeted the chorismate pathway leading to the biosynthesis of pABA from chorismate [27,28]. Wang and coworkers also confirmed the natural status of all of the newly discovered abyssomicins [8]. Recently, abyssomicin B was also isolated from a different marine actinomycete *Verrucosispora* strain MS100047 from the south of China [48].

The structural novelty of abyssomicin C, combined with its biological target and activity, gained considerable interest for biosynthetic evaluation, total synthesis, pharmacological studies, and screening for additional abyssomicin inhibitors. Its challenging molecular architecture has encouraged innovation, which culminated in the discovery of new Diels–Alder synthetic reactions, affording a number of unanticipated abyssomicin analogs, as well as the target abyssomicin C [29]. Many synthetic approaches have centered on the premise of the novel motif deriving from intramolecular trapping of a spirotetronic acid cyclohexene oxide, given the resemblance of the core of abyssomicin C to the latter [29]. The developed synthetic routes have yielded some of the naturally occurring abyssomicins and were also applied for the preparation of new analogues (Section 5).

## 3. Structural Classification and Bioactivity

**Figure 3:**
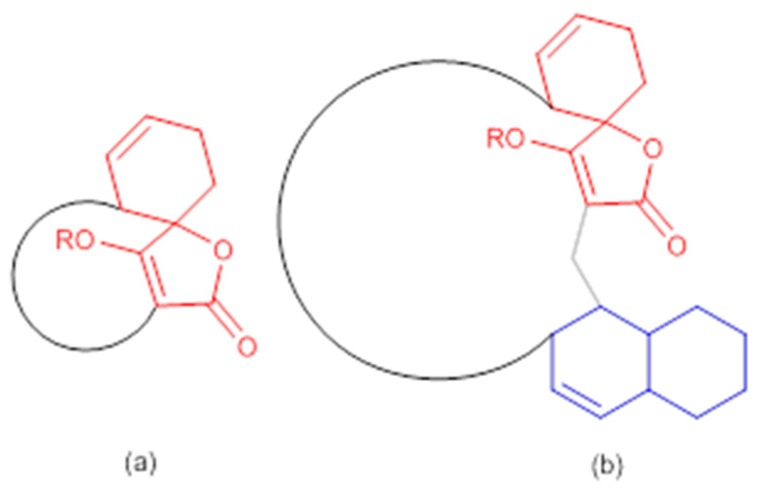
General structures of spirotetronate polyketides (**a**) Class I and (**b**) Class II (modified from [53]). In red: spirotetronate moiety; in blue: decalin moiety.

The abyssomicin family of natural products belongs to the tetronate class of antibiotics, and more precisely, to the subgroup of spirotetronate polyketides [53,54]. Tetronates/spirotetronate polyketides are a relatively new family of microbial metabolites exhibiting mainly antitumor and antibiotic properties [53]. Based on biosynthetic considerations, spirotetronate polyketides are divided into two classes (Class I and Class II). Class II spirotetronates have, in addition to the spirotetronate moiety (with a varying size macrocycle) common to both classes, a decalin moiety (Figure 3) [53]. 

Abyssomicins are considered small class I spirotetronate polyketides (containing an 11-membered macrocycle) [53,54]. The majority of tetronate natural products have been isolated from actinobacteria [7]. Similarly, the abyssomicin family currently contains two related families that derive from actinobacteria of either the genus *Verrucosispora* (abyssomicins B−L) [27,38] or the genus *Streptomyces* (including abyssomicin E, abyssomicin I, ent-homoabyssomicins A and B, abyssomicins 2−5, M–X, and neoabyssomicins A–C) [37,38,49,50,51,55].

**Figure 4:**
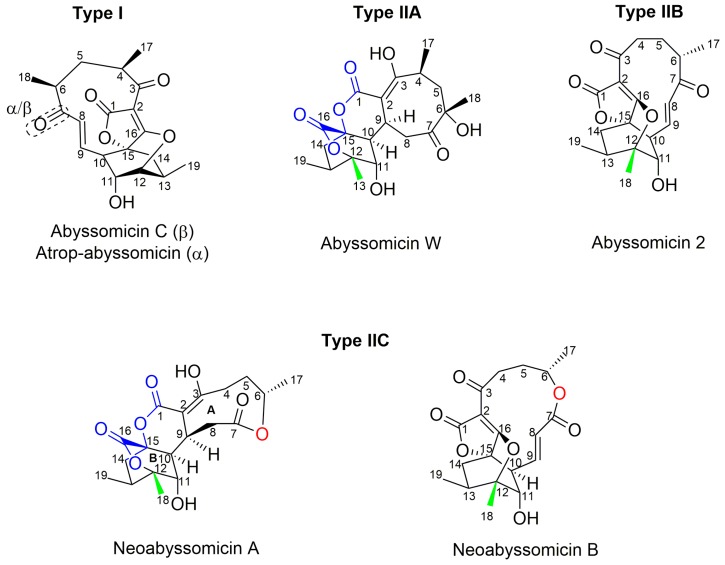
Representative chemical structures of each type and subtype of the abyssomicin class of natural products: type I (abyssomicin C and atrop-abyssomicin C), type IIA (abyssomicin W), type IIB (abyssomicin 2), and type IIC (neoabyssomicin A and B) (modified from [51]).

Apart from classification based on origin, abyssomicins can also be classified according to structure. Abyssomicins are therefore classified into two types (type I and type II) based on their chemically unique scaffold. The type I family includes, to date, abyssomicins B–E, G, H, J–L and atrop-abyssomicin C [51]. Abyssomicins belonging to the type II family are enantiomeric counterparts of the type I family compounds and are further grouped into three subtypes (type IIA, type IIB and type IIC) [51]. Type IIA abyssomicins bear two methyl substitutions (one at C12 and one at C4) while type IIB abyssomicins bear one methyl substitution (at C12) (Figure 4) [51]. Type IIA family members are abyssomicins M–X and ent-homoabyssomicin A and B, whereas type IIB family members include abyssomicin I and 2–5 [51]. The third subtype (type IIC) bears a methyl substitution (at C12) and features an inserted oxygen atom within the polyketide chain. Type IIC includes the dilactone-bridged neoabyssomicins A–D [51] (Figure 4).

Interestingly, the biological activity of abyssomicins is not just restricted to its antimicrobial efficacy against Gram-positive bacteria and mycobacteria. Antitumor properties [37], along with latent human immunodeficiency virus (HIV) reactivator properties [38], anti-HIV properties [51], and HIV replication inducer properties [51], are also reported for this class of compounds. No activity against Gram-negative bacteria or fungi was recorded for any of the natural or synthetic abyssomicins [27,28,34,37]. To date, the antimalarial activity of these agents has not been explored. Some spirotetronates, like kijanimicin, are known to exhibit antimalarial properties in vivo [54], but the mechanism of action responsible for the antiparasitic effect remains unknown [54]. It would be interesting to also explore if abyssomicins, being small spirotetronates [54], exhibit such an antimalarial effect in vitro and in vivo, especially those having an intact Michael acceptor system. However, abyssomicins may only be a valid antimalarial lead, if their mechanism of action underlying the antiparasitic effect is different from that responsible for their antimicrobial effect. In Gram-positive bacteria and mycobacteria, abyssomicins inhibit ADCS (within the chorismate branch of the SCF pathway), inhibiting therefore the production of pABA from chorismate, and ultimately, the production of folate (within the folic acid branch of the SCF pathway) (Figure 1). Nonetheless, the entire SCF pathway is currently debatable as a molecular target in parasites given recent findings [56], which jeopardizes the potential of any SCF inhibitor as an antiparasitic lead. Therefore, even if selectivity of abyssomicins was different between prokaryotes (ADCS) and lower eukaryotes (bifunctional GAT-ADCS), but the molecular target remained within the SCF pathway, abyssomicins would not be valid antimalarial leads. It has been shown in experiments with glyphosate (herbicide inhibitor of the shikimate branch of the SCF pathway), that de novo chorismate synthesis (within the shikimate branch of the SCF pathway) may not be essential for the parasite because parasites are capable of folate salvage [56]. Parasites were shown to predominantly import pABA (rather than pre-formed folates), and other folic/folinic acid substrates, including human folate catabolite pABAGn, through two functional plasma membrane folate transporters (PfFT1 and PfFT2) [56]. This means that the parasite can salvage pathways for its metabolic requirements to survive in the host, even if both the shikimate pathway (leading to chorismate, necessary for pABA production) and chorismate pathway (leading to pABA, necessary for folate production) are blocked (Figure 1) [56]. If abyssomicins were determined effective antiparasitic agents both in vitro and in vivo, the presence of in vivo activity would underline a molecular target different from the SCF pathway (ADCS within the chorismate branch), and would validate the abyssomicin pharmacophore as an antiparasitic lead. On the other hand, if efficacy is only seen in vitro, then the antimicrobial and antiparasitic molecular target of abyssomicins are the same, meaning that the parasite is capable of rescuing metabolic pathway to survive in the host, and abyssomicins would not be valid antiparasitic leads.

Presently, thirty-two natural abyssomicins have been identified, and numerous derivatives were synthesized [27,28,29,30,31,34,35,39,40,41,42,49,50,51,52]. Out of all the abyssomicins screened for antimicrobial activity, only four natural abyssomicins (abyssomicin 2, C, J and atrop-abyssomicin C) [27,28,29,30,35] and nine synthetic derivatives were active (Table 1 and Table 2) [24,31,39,40,41,42,51]. Active abyssomicins against Gram-positive bacteria, including *Micrococcus luteus*, *Bacillus thuringiensis*, *Enterococcus faecalis*, MRSA, and vancomycin-resistant *S. aureus* (VRSA) strains are abyssomicin C and its atropoisomer, 4 atrop-abyssomicin C derivatives (Benzyl ether derivative, Chloro derivative, and two diastereoisomeric methoxymethyl (MOM) ethers derivatives), atrop-O-benzyl-desmethylabyssomicin C, oxidized derivative of abyssomicin I, acetyl abyssomicin C, 3-dithiolane *atrop*-abyssomicin C, and dithiolane abyssomicin C, and abyssomicin 2 (Table 1) [30,31,32,33,34,37,42,51,57]. Active abyssomicins against mycobacteria, including *Mycobacterium smegmatis*, *M. bovis Bacille Calmette Guerin* (BCG), and *M. tuberculosis* are abyssomicin C, atrop-abyssomicin C, and abyssomicin J (Table 2) [35,36]. 

Abyssomicin 2–5, derived from *Streptomyces* RLUS1487, are the first abyssomicins reported as noncanonical reactivators of latent HIV [38]. Among the four abyssomicins isolated, abyssomicin 2 was the most potent latent HIV inducer with an intact Michael acceptor system, while abyssomicin 3 and 4 showed marginal activity [38]. Abyssomicin 2 is the enantiomer of the oxidized derivative of abyssomicin I. Both abyssomicin 2 and I exhibited antimicrobial activity (Table 1) [37,38,52]. Even though abyssomicin 2 was demonstrated as a potent latent HIV inducer, it was shown in a different experiment as highly cytotoxic and having anti-HIV activities [51]. This discrepancy was explained by different virus cell models used in both experiments [51]. Moreover, neoabyssomicins A–C were found to promote HIV-1 viral replication [51].

While the antitumor cell invasion properties were recorded for abyssomicin I and its oxidized derivative at non-cytotoxic concentrations, other abyssomicin derivatives were highly cytotoxic, owing both their activity and toxicity to their active enone (α,β-unsaturated carbonyl) moiety (Table 3) [37,51].

## 4. Mechanism of Action and Binding Site 

### 4.1. Antimicrobial and Antimycobacterial Activity

Abyssomicins are antifolates inhibiting the synthesis of pABA within the chorismate pathway. They irreversibly bind to ADCS via Michael addition to a cysteine residue [30,31,32,33]. 

**Figure 5:**
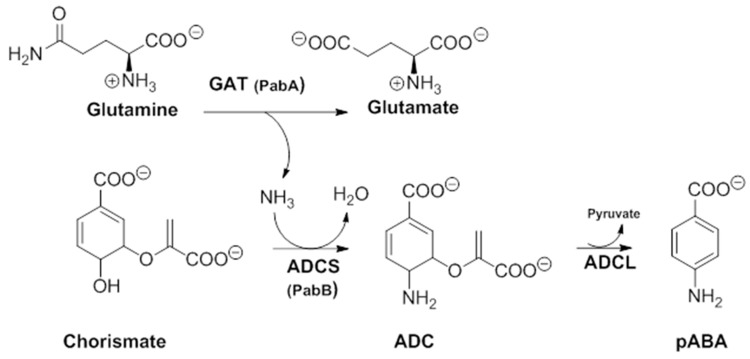
Details of the two-step pathway required for pABA synthesis. In all prokaryotes, the ADC formation requires a bifunctional GAT–ADCS protein, whereas in most prokaryotes, it requires two independent proteins PabA(GAT activity) and PabB (ADCS activity) (modified from [3]). ADC amino-deoxychorismate, ADCL amino-deoxychorismate Lyase, ADCS amino-deoxychorismate synthase, GAT glutamine amido-transferase, pABA para-aminobenzoic acid.

In order to better understand their mechanism of action, it would be useful to first apprehend their molecular target. In a secondary metabolism leading to folates, pABA is synthesized within the chorismate pathway from chorismate in two steps requiring regio-specific amination and aromatization sequences, with overall retention of position and stereochemistry. Chorismate and glutamine are thus aminated by ADCS, yielding glutamate and 4-amino-4-deoxychorismate (ADC). ADC is then aromatized by 4-amino-4-deoxychorismate lyase (ADCL) to generate pABA, with loss of pyruvate (Figure 5). pABA then enters the folic acid pathway, and is added to the pterin moiety by DHPS to later yield active tetrahydrofolates (Figure 1) [9]. The formation of the intermediate ADC in the chorismate pathway requires both a glutamine amidotransferase (GAT) and ADC synthase activity (Figure 5). In many bacteria, such as *Escherichia coli* and *Bacillus subtilis*, ADC synthesis requires two separate enzymes to perform the corresponding enzymatic GAT and ADCS activities. Most prokaryotes possess, therefore, three separate genes encoding the three different enzymatic functions needed for pABA synthesis: PabA-encoded GAT, PabB-encoded ADCS, and PabC-encoded ADCL activities (Figure 5) [3,8,59,60], whereas plants and lower eukaryotes possess two genes encoding two separate enzymes: a bifunctional glutamine amidotransferase–aminodeoxychorismate synthase (GAT–ADCS) enzyme and an ADCL enzyme [1,3,8,24,61,62]. 

**Figure 6:**
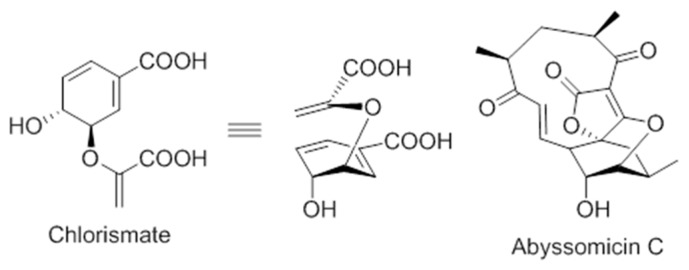
Comparison of the conformations of chorismate and abyssomicin C [63].

On the structural basis, the oxabicyclooxane ring system of abyssomicin C and atrop-abyssomicin C show striking similarity to the chorismate transition state analogue (Figure 6), suggesting, therefore, that these compounds are substrate mimetics [32].

**Figure 7:**
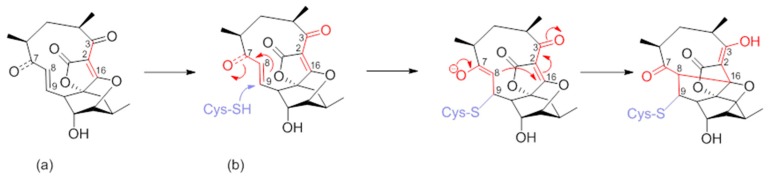
(**a**) Atrop-abyssomicin C; and (**b**) irreversible inhibition of PabB via double Michael addition (Michael acceptors in red) (modified from [63]).

Numerous synthetic and chemical biology efforts were combined to decipher the mechanism of action of abyssomicins at the molecular level. Studies confirmed that abyssomicin C and atrop-abyssomicin C are substrate mimetics that irreversibly bind the Cys263 of the PabB subunit of ADCS (*B. subtilis* and *E. coli*) in a Michael addition-based enzyme-trapping mechanism, forming a sulfur-bound abyssomicin d-like structure in the process (Figure 7b) [31,33,34,39,40,43,45,63]. This rearrangement into a pentacyclic abyssomicin D-like structure takes place through a sequential reaction of thiol addition/cyclisation, where the Cys-263 amino acid from active abyssomicins acts as an S-nucleophile, and binds covalently to the ADCS by exploiting the reactivity of the conjugated ketone functionality at C9. The initial attack of Cys-263 onto the conjugated ketone produces a transiently formed C-8 nucleophile, and this process is followed by Michael addition of the generated enolate/enol onto the tetronic moiety (C-8 nucleophile reacts with the spirotetronate subunit at the C-16 center), irreversibly binding to ADCS and affording, as a final product, a pentacyclic derivative of abyssomicin D (Figure 7b) [34,63]. As a result, subsequent biosynthesis steps from the branch-point metabolite chorismate are inhibited [27,28,64]. This mechanism is attributed to the α,β-unsaturated ketone, which is not present in inactive abyssomicins [31,32,33,39,40,44,45]. The protein-binding site of atrop-abyssomicin C on the correspondent amino acid side chain of the PabB subunit was determined to be the thiol side chain of Cys263 of the peptide TPDFQIICGSPE, located at the proximity of the active site of PabB [30,31,32,33]. 

Notably, abyssomicin J (Figure 2) is the first natural prodrug for atrop-abyssomicin C, undergoing reverse Michael addition, in vivo, by means of in situ enzymatic oxidation via the P450 enzyme to yield atrop-abyssomicin C [35]. 

### 4.2. Viral Induction

Highly active antiretroviral therapy (HAART) has been effective in decreasing active viral loads in HIV patients, but HAART is not curative, and its discontinuation results in viral rebound and disease progression. Since the persistence of latent viral reservoirs prevents eradication of HIV, a promising strategy to achieve a cure for HIV is to reactivate the latent provirus in combination with HAART. The deliberate induction of viral replication from its latent state is proposed to eliminate HIV-harboring cells either by direct viral cytopathic effects or by rendering those cells susceptible to immune system regulation [65,66]. One approach for latent viral induction employs pharmacological modulators of signaling pathways associated with viral reactivation. Published reactivating agents have been predominantly limited to histone deacetylase (HDAC) inhibitors, agonists of protein kinase C (PKC) and agonists of transcription elongation factors [38]. The abyssomicins represent a novel structural class of reactivating agent with ex vivo activity through an HDAC and PKC-independent mechanism, making them intriguing from a mechanistic perspective [38]. The precise mechanism of action inducing latent HIV virus is yet to be determined. Moreover, the HIV viral replication and anti-HIV mechanism of action of some abyssomicins are currently under investigation [51]. 

## 5. Isolation and Syntheses of Novel Abyssomicins

### 5.1. Synthesis of Abyssomicin B–D

**Figure 8:**
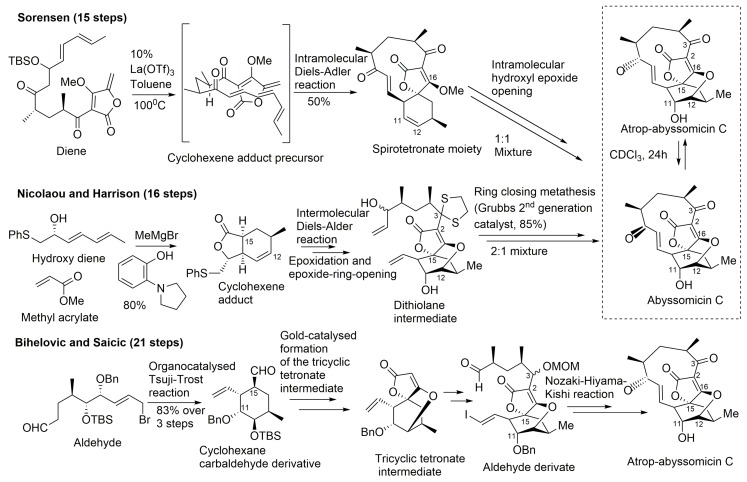
Highlights of the total synthesis of abyssomicin C (by Sorensen and Nicolaou, and their coworkers) and of the total synthesis of atrop-abyssomicin C (by Bihelovic and Saicic) (modified from [41,53]).

To date, two successful total synthesis of abyssomicin C have been reported, the first by Sorensen and coworkers, and the second by Nicolaou and Harrisson (Table 1, Figure 8) [31,32,33,39,43]. Furthermore, numerous attempts have been reported, such as those of Couladouros and coworkers, and those of Snider and Zou, which were also considered as formal syntheses [33,40,43,44,45]. 

A common feature to all these retrosynthetic concepts is the use of a common retron, the central cyclohexane core, for the application of the Diels–Alder reaction (Figure 8). The syntheses by Sorensen and coworkers, and Nicolaou and coworkers, both rely on a Diels–Alder reaction as the key step in the construction of the oxabicyclooctane core. Both synthetic approaches highlight, therefore, the power of the Diels–Alder reaction, either intramolecular, to form a strained macrocyclic system (Sorensen and coworkers), or intermolecular, via a Lewis acid templated transition state (Nicolaou and coworkers) [33,40,43,44,45,57]. The Sorensen synthesis featured a presumed biomimetic late stage intramolecular Diels–Alder reaction providing the tricyclic system of their target. This was followed by a short sequence of reactions involving stereoselective epoxidation of the cyclohexene double bond and hydroxyl epoxide opening [39,45]. 

The target molecule was synthesized by a convergent approach with a longest linear sequence of 15 steps (overall yield: 0.9–1.7%) [43]. Atrop-abyssomicin C was transformed into abyssomicin C under mild acidic conditions (Figure 8). 

Nicolaou and coworkers adopted an alternative synthesis featuring an intermolecular Diels–Alder approach followed by an epoxidation and an epoxide ring-opening sequence to form the oxabicyclooctane core structure intermediate (dithiolane abyssomicin C) [31,33,40,43,44,57]. A ring-closing metathesis reaction is projected as the means to forge the strained 11-membered ring of the abyssomicin skeleton (note S6, supplemental data) [43,57]. The longest linear sequence in this convergent strategy consisted of 16 steps (overall yield: 3.4%) (Figure 8) [43]. The Nicolaou and coworkers strategy led to a 2:1 mixture of abyssomicin C and atrop-abyssomicin C under mild acidic conditions [43], whereas the Sorensen synthesis afforded, in mild acidic conditions, a 1:1 mixture of the target molecule abyssomicin C with atrop-abyssomicin C (primarily dubbed as iso-abyssomicin C) (Figure 8) [29,33,40]. Atropoisomers were separated by column chromatography [43]. The conditions of the final step in the Sorensen synthesis of abyssomicin C were demonstrated later by Nicolaou and coworkers to affect the equilibration of the abyssomicin C atropisomers [31,33,40,43].

Concurrent with Sorensen’s first total synthesis of abyssomicin C, Snider and Zou disclosed a related intramolecular Diels–Alder approach towards that active compound by constructing an advanced intermediate and attempting to convert it into the target molecule [44]. Although unsuccessful in yielding abyssomicin C, they provided facile access to the carbocyclic skeleton of abyssomicin C through the same advanced intermediate synthesized in Sorensen’s lab, which rendered their work as a formal total synthesis [44]. Conversely, the work of Snider and Zou unveiled a compound possessing the abyssomicin D carbon skeleton. Their strategy consisted of 6 steps to yield the intramolecular Diels–Alder substrate trienyl methylenebutenolide by coupling trienone keto aldehyde with 3-methoxy-4-methylenebutenolide. Heating the latter in CHCl_3_ for 2 d at 70 °C afforded 80% of the same Diels–Alder intermediate synthesized in Sorensen’s lab, having the complete carbon skeleton of abyssomicin C. Addition of thiophenoxide to the enone double bond of the Sorensen intermediate, followed by an intramolecular Michael addition, afforded a substrate with the abyssomicin D carbon skeleton [44]. This was the first synthetic entry into the abyssomicin D ring framework, and it provided experimental support for the proposed biosynthesis of abyssomicin D [31,33,40,44].

Shortly after this report, Couladouros and coworkers published their formal total synthesis of abyssomicin C that also proceeded through the same Diels–Alder strategy. During their approach, the use of excess of iodine resulted in the formation the abyssomicin D carbon skeleton in a similar manner that Snider and Zou observed for a sulfide analogue [31,33,40,44,45].

It also seems that the synthesis of abyssomicin D is possible from both atrop-abyssomicin C and abyssomicin C with an NADH analogue, and with NaBH_4_ in THF, respectively [30]. Additionally, Bihelovic and coworkers also reported a formal synthesis of abyssomicin B and D note S7, supplemental data) [34,41,58].

### 5.2. Isolation and Synthesis of Atrop-Abyssomicin C

En route to abyssomicin C, Nicolaou and Harrisson prepared and characterized its stable conformational isomer atrop-abyssomicin C, which in the presence of a strong acid, underwent an interconversion into abyssomicin C [31,33,39,40]. Atrop-abyssomicin C is, therefore, the genuine secondary metabolite, while the originally described abyssomicin C is a conformational artifact (Figure 8) [34]. It also seemed as if this mechanistic interconversion can be brought about under numerous controlled conditions, including those adopted by Sorensen and coworkers, who also reported the identification of atrop-abyssomicin C, which they termed iso-abyssomicin C, in the final step of their successful total synthesis of abyssomicin C [31,33,40]. Under mild acidic conditions, the Nicolaou and coworkers strategy afforded a 2:1 mixture of abyssomicin C and atrop-abyssomicin C, while that ratio was 1:1 in the Sorensen synthesis (note S8, supplemental data) [29,33,40]. Reconsideration of experimental data from previous cultivations of *Verrucosispora* AB-18-032 confirmed the presence of natural atrop-abyssomicin C that was previously described by synthetic efforts only (note S9, supplemental data) [30]. 

Bihelovic and coworkers reported in 2012 an enantioselective total synthesis of (−)–atrop–abyssomicin C by a route that allowed the preparation of analogues for further SAR studies (note S7, supplemental data). The Bihelovic synthesis of abyssomicin natural products did not rely on the Diels–Alder reaction, which has been extensively used by the other groups mentioned above. In their ingenious strategy, the synthesis of atrop-abyssomicin C was based on a dual organotransition metal catalysis process, and encompassed 21 steps grouped in three main stages: (1) the dual catalysis formation of the functionalized key cyclohexane core with all stereocenters installed using a newly developed cyclization method by an organocatalyzed Tsuji–Trost reaction (combining organocatalysis and Pd-catalyzed allylation processes) [34,41,67,68,69,70,71,72]; (2) the formation of the tricyclic core by a gold-catalyzed reaction sequence that allowed a nucleophilic attack of a β-hydroxy group onto a tetronate motif, instead of the alternative route involving ring opening of an epoxide by the tetronate [41]; and (3) macrocyclization through the attachment of the side chain and completion of the synthesis by an eleven-membered ring closure by the Nozaki–Hiyama–Kishi reaction (note S10, supplemental data) (Figure 8) [41]. 

### 5.3. Synthesis of Atrop-o-Benzyl-Desmethylabyssomicin C

In 2014, Matovic and coworkers reported the total synthesis of atrop-*o*-benzyl-desmethylabyssomicin C, an atrop-abyssomicin C analogue. The synthesis of atrop-*o*-benzyl-desmethylabyssomicin C was accomplished using diastereotopos-selective ring closing metathesis and Nozaki–Hiyama–Kishi cyclization as the key steps. The atrop-abyssomicin analogue was active, and three-fold less toxic compared to atrop-abyssomicin C [42]. 

### 5.4. Isolation of Abyssomicin E

In 2007, Niu and coworkers isolated abyssomicin E from a Senegalese *Streptomyces* sp. HKI0381 taken from a soil sample in Ile de Paradis. They were able to determine its structure by comprehensive nuclear magnetic resonance (NMR) and mass spectrometry (MS) analysis. For the first time in this recently discovered class of abyssomicins, the absolute stereochemistry was directly established by subsequent single-crystal X-ray (Figure 2) [55]. The absolute stereochemistry of abyssomicin E was in accordance with the configurations of abyssomicins B–D that were determined earlier indirectly by the Mosher and Helmchen methods, and were later confirmed by total synthesis (Figure 2) [55]. 

### 5.5. Isolation and Synthesis of Abyssomicin G and H

After the isolation of abyssomicin B–D in 2004 from *Verrucosispora sp*. AB-18-032, a reinvestigation of the culture filtrates from fermentations of the same species conducted by Sassmuth and coworkers in 2007 led to three additional co-metabolites in the form of atrop-abyssomicin C, abyssomicin G, and abyssomicin H. Nomenclature of the new compounds was based on the historic course of the abyssomicin research [30]. Wang and coworkers also confirmed the natural product status of the abyssomicin co-metabolite H [35]. The synthesis of abyssomicin H from atrop-abyssomicin C was possible with NaBH_4_ in THF [30]. The total synthesis of abyssomicin H along with a formal synthesis of abyssomicin G has also been reported by the Bihelovic team in their enantioselective total synthesis of (−)-atrop–abyssomicin C using an approach based on Pd-catalyzed alkylation and Nozaki–Hiyama–Kishi reaction macrocyclization. The pivotal steps in the synthesis were stereoselective formation of the cyclohexane core, formation of tricyclic spirotetronate intermediate, and the eleven-membered ring closure (note S7, supplemental data) [34,41].

### 5.6. Isolation of Abyssomicin I and Synthesis of Its Derivatives

In 2010, the *Streptomyces* CHI39 strain isolated from a rock soil sample in Campeche, Mexico, was found to produce abyssomicin I. This new member of the unique spirotetronate polyketide family was the sole abyssomicin detected from the investigated sample [37]. The structure of abyssomicin I was elucidated by Igarashi and coworkers [30]. Abyssomicin I (Figure 2) is a new abyssomicin variant derived from a different methylation incorporation pattern within the polyketide biosynthesis pathway [30]. Chemical derivatization, and application of the modified Mosher method, yielded nine different derivatives of abyssomicin I, of which only the oxidized derivative exhibited antimicrobial activity (Table 1). The synthetic oxidized derivative of abyssomicin I resulted from a selective oxidation (using manganese dioxide) of the C7 secondary allylic hydroxyl group to a ketone, which restored the Michael acceptor lacking in abyssomicin I [37].

### 5.7. Isolation of Ent-Homoabyssomicin A and B

In 2011, Abdalla and coworkers reported the isolation and characterization of two novel members of the abyssomicin family ent-homoabyssomicin A and B (Figure 2) from a forest soil *Streptomyces* sp. strain (Ank 210) obtained from Kaiserslautern in Germany [49]. The absolute stereochemistry of the first compound was assigned by single-crystal X-ray diffraction. The nomenclature ent-homoabyssomicin was suggested because its configuration (2S, 4S, 6S, 8S, 10S, 11S, 12S, 13S, 15S, 16R) appeared enantiomeric to that of abyssomicin D (4R, 11R, 12R, 13R, 15R) [49].

### 5.8. Isolation and Semi-Synthesis of Abyssomicins J–L

In their search for anti-tubercular compounds (note S11, supplemental data), Wang and coworkers reported in 2012 the isolation of three new abyssomicins along with the four known abyssomicins B, C, D, and H from a *Verrucosispora* strain (MS100128) isolated from deep-sea sediment of the South China Sea [35]. Structures (Figure 2) were assigned to the new abyssomicins J, K, and L by detailed spectroscopic analysis. Based on biogenetic grounds, abyssomicins J–L were shown to be co-metabolites of abyssomicins B–D and atrop-abyssomicin C, biosynthetically related and likely derived from abyssomicin C. Their semi-synthesis from the co-metabolite abyssomicin C confirmed this biosynthetic relationship [35]. 

Abyssomicin J, K, and L were each semi-synthesized separately from abyssomicin C in reaction with Na_2_S (note S12, supplemental data), NaOH (note S13, supplemental data) (or TFA with/without NaOH) (note S14, supplemental data), and MeOH (or TFA in MeOH) (note S15, supplemental data), respectively [35]. While abyssomicin K and L possess structural properties found in congeners E and D, abyssomicin J had a unique dimeric structure and was demonstrated to serve as a prodrug, selectively delivering atrop-abyssomicin C (note S16, supplemental data) upon oxidative activation [35]. In addition, the Bihelovic laboratory reported a formal synthesis of abyssomicins J–L in their successful attempt to synthesize atrop-abyssomicin C (note S7, supplemental data) [34,41].

### 5.9. Isolation of Abyssomicin 2–5

The screening of a marine natural products library for selective reactivators of latent HIV virus led Leon and co-workers to the identification of abyssomicins 2–5 from the marine-derived *Streptomyces* sp. RLUS1487 strain as novel activators of latent HIV. Further investigations unveiled that abyssomicin 2 is the enantiomer of the oxidized derivative of abyssomicin I while abyssomicin 3–5 constitute three novel abyssomicin analogues (Figure 2) [38].

### 5.10. Isolation of Abyssomicin M–X

In 2017, Wang and coworkers isolated 12 new enantiomeric-like abyssomicin analogues (abyssomicins M–X) from the coalmine fire isolate *Streptomyces* sp. LC-6-2 in Perry County, Kentucky, United States [50]. The isolation of abyssomicins M–X was the first report of abyssomicins sharing global stereochemical features with the unique enantiomeric ent-homoabyssomicins A and B [50]. The structures of abyssomicins M–X were elucidated. Notably, abyssomicin W contains an unprecedented 8/6/6/6 tetracyclic core with C2–C16 disconnection and C2–C9 connection unique among abyssomicins reported to date (Figure 2) [50]. Moreover, the bicyclic abyssomicin X represents the first reported naturally occurring linear spirotetronate, which expands the structural diversity of abyssomicin-associated scaffolds reported to date (Figure 2) [50]. 

### 5.11. Isolation of Neoabyssomicins A–C and Semi-Synthesis of Neoabyssomicin D

Neoabyssomicins A–C were isolated in 2017, along with the previously described abyssomicins 2 and 4, from the deep-sea derived *Streptomyces koyangensis* SCSIO 5802 in South China. Neoabyssomicin B was the main product of the strain [51]. Neoabyssomicins A–C constitute a new subtype (type II_C_) of abyssomicins, featuring a methyl substitution at C12 and an oxygen atom inserted in the polyketide chain and possess unique structures (Figure 2 and Figure 4) [51]. For instance, neoabyssomicin A is formed by a novel skeleton featuring a rare caged 6/6/6 ring system fused with two additional lactones (Figure 4) [51], while neoabyssomicin B has a 12-membered lactone ring in place of the 11-membered polyketide ring, with neoabyssomicin C being its seco-form (Figure 2) [51]. Treatment of neoabyssomicin B with methanol/H_2_O/acetic acid (10:90:0.1) at 90 °C for 1 d yielded neoabyssomicin D [51]. 

## 6. Biosynthesis and Interconversion of Abyssomicins

Natural abyssomicins derive from actinobacteria of either the genus *Verrucosispora* (abyssomicins B−L) [27,38] or the genus *Streptomyces* (abyssomicin E, I, 2–5, ent-homoabyssomicins A and B) [18,67,68,69]. Atrop-abyssomicin C, followed by abyssomicin B, seem to be the main products of fermentation of the marine-derived *Verrucosispora* AB-18-032, whereas abyssomicins C, D, G, and H are minor congeners [30,31,34]. Abyssomicins B–D, J–L, and atrop-abyssomicin C are co-metabolites of fermentation of the *Verrucosispora* MS100128 strain [35]. On the other hand, abyssomicin E, abyssomicin I, ent-homoabyssomicins A and B, abyssomicins 2–5, M–X, and neoabyssomicins A–C derive from different *Streptomyces* strains (HKI0381, CHI39, Ank210, RLUS1487, LC-6-2, and SCSIO 5802, respectively) [37,38,49,50,51,55]. 

Abyssomicins are small tetronates belonging to the Class I spirotetronate polyketides [53,54]. Natural abyssomicins are thought to be biosynthesized in a similar fashion to the biosynthesis of kijanimicin-type spirotetronate compounds where a polyketide biosynthetic pathway, followed by an intramolecular Diels−Alder reaction between the diene part in the polyketide chain and the exo-methylene dienophile in the tetronic acid moiety, yield a polycyclic abyssomicin framework [37].

Recent elucidation of the biosynthetic gene clusters of tetronic acid-containing antibiotics (chlorothricin, kijanimicin, tetronomycin, and tetrocarcin) suggests that five conserved genes play a role in tetronic acid biosynthesis [73]. The biosynthetic gene cluster (*aby*) of abyssomicin C spans 57 kb and 24 open reading frames (ORFs) [51,73]. 

The abyssomicin C gene cluster (*aby*), isolated from *V. maris*AB-18-032 [73], comprises the anticipated polyketide synthase I (PKS I) genes (*abyB1*, *abyB2*, and *abyB3*) and five genes (*abyA1–A5*) involved in the assembly of the tetronic acid moiety, three genes encoding oxygenases (*abypE*, *abyX*, and *abyV*), as well as genes with putative regulatory, export, and possibly import functions (*abyI*, *abyH*, *abyC*, *abyD*, *abyR*, *abyI*, *abyT*, and *abyF1*–*abyF4*). The function of *abyK* gene remains obscure [72]. The presence of these five genes *abyA1*–*A5* involved in the assembly of the tetronic acid moiety in the abyssomicin gene cluster and their homology to the chlorothricin biosynthetic gene cluster (*chlM* and *chlD1*–*4*) having the same function strengthens this hypothesis [73].

**Figure 9:**
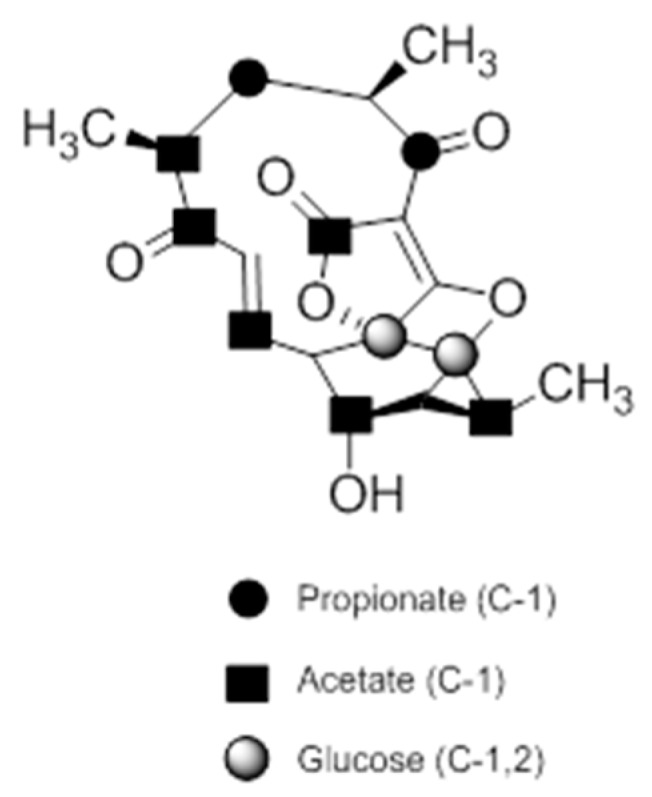
Incorporation of ^13^C-labelled precursors into the polyketide backbone of abyssomicin C (modified from [73]).

**Figure 10:**
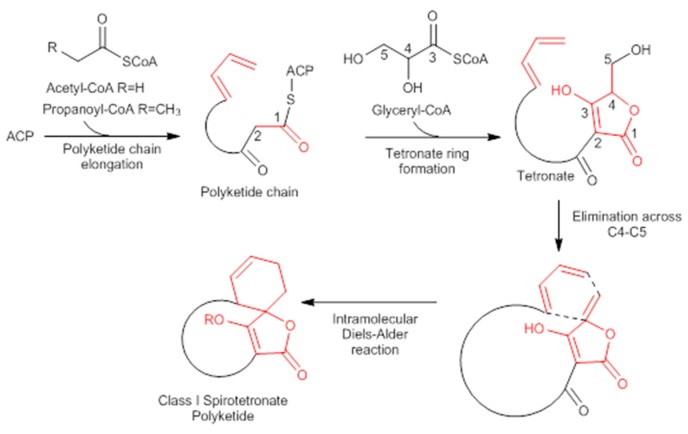
Biosynthesis of Class I spirotetronate polyketides (modified from [53]). ACP acyl carrier protein.

A combination of feeding studies with ^13^C-labelled biosynthetic precursors on cultures of *Verrucosispora maris* AB-18-032 were conducted, and the abyssomicin biosynthetic gene cluster of abyssomicin C was identified. Based on the findings from the aforementioned studies, the atrop-abyssomicin biosynthesis is found to be initiated via the type I polyketide synthase pathway [53] as a linear polyketide chain is formed (from five acetates, two propionates, and a metabolite from the glycolytic pathway) (Figure 9) [53,54,73]. The construction of the polyketide chain starts by condensation of acetic acid units, and proceeds by elongating their carbon chain via attachment of acetyl and/or propanoyl units (*abyB1*, *abyB2*, and *abyB3*) to the acyl carrier protein (ACP) (*abyA3*) by the acyl transferase (*abyA2*). The polyketide synthase processing is followed by the formation of tetronate by incorporation of a glyceryl unit, via glyceryl-CoA (by a Claisen condensation followed by lactonization). The tetronate formation is followed by an elimination of the hydroxy group at C5 via acetylation and subsequent elimination, thereby forming dienophile [53]. An intramolecular Diels–Alder reaction then generates the characteristic Class I spirotetronate polyketide moiety (Figure 10).

The resulting substrates subsequently undergo peripheral oxidations. The epoxide formation catalyzed by the oxygenase (*abyE*) is followed by ring opening to yield atrop-abyssomicin C [53,54,73].

The neoabyssomicins A–C/abyssomicin 2,4 biosynthetic gene cluster (*abm*), isolated from *S. koyangensis* SCSIO 5802 [52,56], spans 62.9 kb and 28 ORFs [40]. Similarly to the abyssomicin C gene cluster (*aby*) [73], *abm* comprises the PKSI genes (*abmB1*–*B3*, homologous to *abyB1*–*B3* in *aby*) coding for the assembly of the neoabyssomicins A–C/abyssomicin 2,4 polyketide backbone and five consecutive genes (*abmA1*–*A5*, homologous to *abyA1*–*A5* in *aby*) coding for tetronate biosynthesis [52]. The *abm* biosynthetic gene cluster also comprised the *abmU and abmV* genes associated with the biosynthetic Diels–Alder chemistry and oxygenation, respectively; as well as *abmD*, *abmF1*–*F4* genes implicated in transport functions and *abmI* and *abmH* involved in regulatory functions. Moreover, *abmI* and *abmH* represent upstream and downstream boundaries of *abm*, acting as pivotal positive regulators of neoabyssomicins A–C/abyssomicin 2,4 biosynthesis [52]. abmU, was proposed to catalyze this intramolecular spirocyclization. All the aforementioned genes showed homologies (up to 75%) to corresponding counterparts in the atrop-abyssomicin C *aby* cluster [52]. Nonetheless, seven genes in *abm* (*abmK*, *abmL*, *abmM*, *abmN*, *abmJ*, *abmG*, and *abmE2*) have no apparent homologous counterparts in the *aby* cluster [52]. *abmV*, *abmM*, *abmJ*, *abmG*, *abmE1*, and *abmE2* genes are all predicted to encode enzymes related to oxidation or reduction, and are therefore excellent tailoring enzyme candidates for the biosynthesis and interconversion [52]. However, although their involvement in these conversions is highly likely, the precise details for how these gene products carry out their relevant chemistries remains to be determined [52].

Neoabyssomicin/abyssomicin (neoabyssomicins A–C/abyssomicin 2,4) polyketide biosynthesis is initiated via the type I polyketide synthase pathway [52,53], similarly to atrop-abyssomicin C [52,53]. Three consecutive type PKSI genes (*abmB1*, *abmB2*, and *abmB3*) encode a total of seven PKS modules in the *abm* cluster for the assembly of the neoabyssomicin/abyssomicin polyketide backbone. The formation of the polyketide backbone is initiated when the activated ketosynthase domain of *abmB1* acts as a loading module for formation of the acetate starter unit by catalyzing decarboxylation of an ACP-tethered malonate [52]. Chain elongation proceeds via attachment of acetyl and/or propanoyl units via malonyl-CoA (*abmB1*, *abmB2*, and *abmB3*). The polyketide synthase processing is followed by the formation of tetronate (*abmA1*–*A5*) (note S17, supplemental data) [52]. The Diels–Alderase, *abmU*, catalyzes the intramolecular spirocyclization to form the spirotetronate skeleton [52]. 

In contrast with other natural abyssomicins having a biosynthetic origin similar to that of atrop-abyssomicin C [49], abyssomicin I (isolated from the *Streptomyces* CHI39 strain) is thought to be a novel variant of abyssomicins, with a different methylation pattern previously not described [37]. Although its tetracyclic skeleton is similar to that of abyssomicin C and G, abyssomicin I is thought to derive from a different incorporation pattern of extender unit, where the position of methyl branching is determined by the acyltransferase domain in polyketide synthases, which recruits methylmalonyl-CoA for chain elongation [37].

Little is known about the mechanism of interconversion of abyssomicins, but the work conducted, to date, on these polyketides shows that abyssomicin G [30] and abyssomicin C [27,33,40] are precursors of abyssomicin B, with abyssomicin C being its direct precursor [30]. Furthermore, abyssomicin C is a precursor of abyssomicin D. Moreover, atrop-abyssomicin C serves as the direct precursor of abyssomicin D [27,29,31,33,39,40] and H [30]. Abyssomicin B was proposed to derive from abyssomicin C via oxidative 1,4-addition of hydroxylamine, and subsequent ring closure by oxidation [27,29,33,40]. It was speculated that the α/β-unsaturated ketone, lacking in the inactive abyssomicins (B and D), serves as a Michael acceptor, and is involved not only in the mechanism of action of the active abyssomicin C, but also in its biotransformation into both abyssomicin B and D (Figure 2) [33]. The interconversion of abyssomicin D and abyssomicin H from atrop-abyssomicin C and/or abyssomicin C is achieved via a formal hydride addition [30,31]. Incubations of abyssomicins C and atrop-abyssomicin C with NaBH_4_ in THF led to the formation of both D and H [30]. The incubation of atrop-abyssomicin C with NADH analogue yields abyssomicin D, confirming, therefore, these assumptions [30]. Given that abyssomicin D is a 6,9-deoxy analogue of abyssomicin E, it can therefore be reasoned that the mechanism of interconversion from abyssomicin D to abyssomicin E requires enzymatic oxygenation of the polyketide skeleton at C-6. Additionally, the biosynthesis of abyssomicin G is speculated to occur via addition of ammonia and subsequent *N*-oxidation to the abyssomicin core. *N*-oxidized natural products have been found to be often biosynthesized via a hydroxylamine intermediate catalyzed by flavin monooxygenases, which are expected to be involved in the biosynthesis of abyssomicin G [30]. Moreover, abyssomicins J–L could be produced as Michael addition adducts of abyssomicin C [35], and are therefore believed to be, similarly to abyssomicin B, D, G, and H, products of post-transformation of abyssomicin C [34]. Abyssomicin C, along with *Streptomyces*-derived abyssomicin E and I, are thought to be produced via the genuine polyketide biosynthesis pathway [34]. Neoabyssomicins B seems to be the main product of fermentation of *Streptomyces koyangensis* SCSIO 5802 [51]. It was proposed that abyssomicin 4 serves as an early biosynthetic intermediate of abyssomicin 2 and neoabyssomicins A–C [51,52]. A C9 dehydration of abyssomicin 4 is thought to yield abyssomicin 2 [51,52]. A Baeyer–Villiger oxidation of the latter is believed to give neoabyssomicin B [51,52]. The newly formed C7 lactone group in neoabyssomicin B can readily suffer under mild hydrolysis conditions to yield neoabyssomicin C, or methanolysis to yield neoabyssomicin D [51]. Neoabyssomicin A can be biosynthesized from neoabyssomicin B through a sequence of hydrolysis at C16, keto–enol tautomerism, a retro-aldol reaction (to establish the C16 containing lactone), establishment of the C2–C9 linkage via an aldol-type Michael reaction, and finally, followed by a simple keto–enol tautomerization [51,52]. Nonetheless, failure to synthesize neoabyssomicin A from neoabyssomicin B in the lab indicates that such a biosynthesis may require some level of enzymatic involvement [51,52]. Indeed, the tetronate moieties result from an enzymatically driven acetylation-elimination sequence, accomplished by an acyltransferase E2 component of 2-oxoacid dehydrogenase multienzymes (*abmA4*) and an α/β hydrolase fold protein (*AbmA5*) [52].

## 7. Structure–Activity Requirements

### 7.1. Antimicrobial and Antimycobacterial Activity

**Figure 11:**
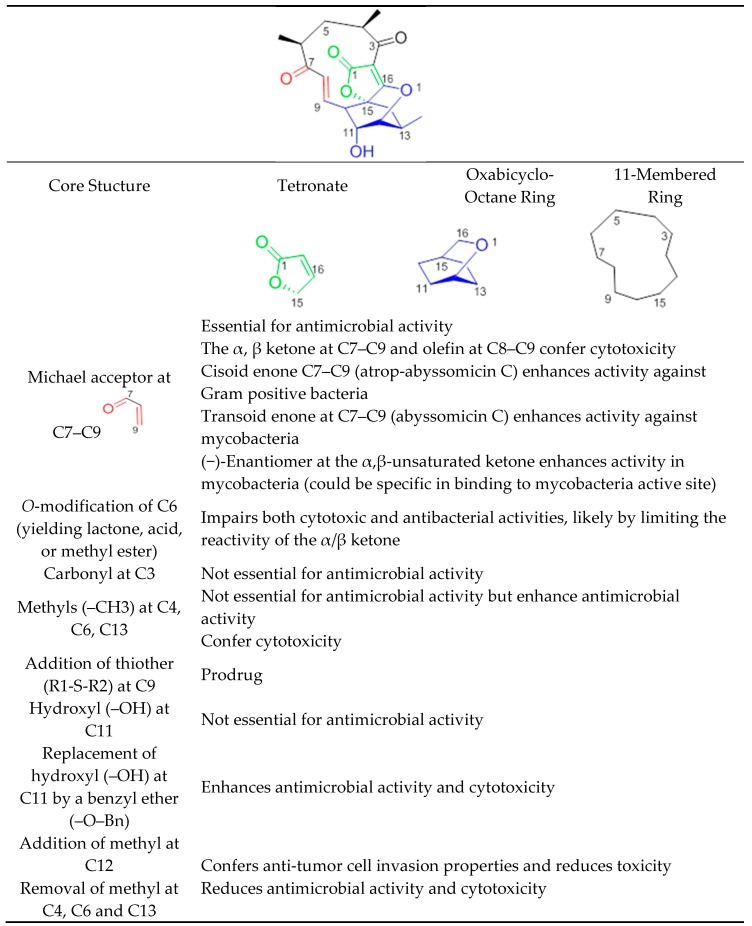
Structure–activity relationship of abyssomicins conferring antimicrobial activity against Gram-positive bacteria and mycobacteria.

Abyssomicins possess an intriguing molecular assembly by virtue of their unique combination of structural motifs. They are class I spirotetronate-polyketides [53]. Common structures of this class of compounds include an oxabicyclooctane system (four interwoven ring cycles), an 11-membered ring carrying an α,β-unsaturated ketone (or a hydroxyl in the case of abyssomicin B and I), and a tetronate moiety (Figure 11) [33]. The α,β-unsaturated ketones function adjacent to their oxabicyclooctane ring, and along with a trans-configured olefin, form a Michael acceptor system, located at C7–C9, that is essential to exert antimicrobial activity (Figure 11). Missing activities of abyssomicin B, D, E, G, H, I, K, L, M–X, ent-homoabyssomicin A and B, due to the reduction of the alkene to the corresponding alkane at C8–C9 (abyssomicin B, D, E, G, H, K, L, M–X, ent-homoabyssomicin A, B, neaoabyssomicin A), and the reduction of the ketone in C7 to a hydroxyl (abyssomicin B and I), destroys the Michael acceptor enone moiety. This further validates the importance of an intact Michael acceptor system as a SAR requirement for abyssomicins [27,28,30,32,50,51]. This intact Michael acceptor system is thought to be involved not only in the mechanism of action of active abyssomicins but also in the biotransformation and biosynthesis of the members of this family, as described previously (Section 6). Moreover, missing activities of the linear abyssomicin X, emphasize the role of cyclic structure in conferring both activity and toxicity [51], while missing activity of neoabyssomicins A–C emphasizes the role of an intact 11-membered ring in conferring antimicrobial activity [51]. 

The presence of such a reactive enone moiety in organic molecules can also give rise to cytotoxicity (Table 3) [34]. *o*-Methylation at C6, impairing both cytotoxic and antimicrobial activities, limits the reactivity of the enone, and further validates this observation [51]. The cytotoxicity of enones is presumed to be the consequence of non-selective alkylation of cellular nucleophiles, while the antimicrobial activity has been correlated with the ability of the Cys-nucleophile of the reactive Michael acceptor system to attack and covalently bind ADCS [42]. Compared to other active abyssomicins (Section 4.1), atrop-abyssomicin C is considered to be a more potent Michael acceptor, and is therefore expected to exhibit a more potent antibacterial activity (Table 1). However, the efficacy of the active abyssomicin compounds against different bacterial targets seems to be a definite structural constraint, suggesting the requirement of structural features other than the presence of α,β-unsaturated carbonyl systems [36]. In that regard, it was reported that abyssomicin C is more effective against some mycobacterial strains, whereas atrop-abyssomicin C was found to be more effective against Gram-positive bacteria, including MRSA (Table 1 and Table 2). The cisoid enone at C7–C9 of atrop-abyssomicin C exhibits a higher degree of planarity as compared with the transoid enone at C7–C9 of abyssomicin C, suggesting that atrop-abyssomicin C should exhibit a higher degree of conjugation and, therefore, higher reactivity (note S18, supplemental data). This observation is true in Gram-positive bacteria [27,28,29,30,31,32,33,34,37,42,57] but not in mycobacteria [35,36]. The slightly improved activity of atrop-abyssomicin C against Gram-positive is attributed to atropisomerism, and increased reactivity toward nucleophiles when compared with abyssomicin C [33]. On the other hand, the positioning of the potentially reactive α, β-unsaturated ketone in the 11-membered macrocyclic ring may constitute an SAR requirement for efficacy against mycobacteria [36]. (−)-Abyssomicin C and (−)-atrop-abyssomicin C had slightly improved activity against mycobacteria when compared to their respective (+)-antipodes (Table 2) [36], which points towards some specificity in mode of action in mycobacteria, supporting inhibition through a specific binding event [36]. In addition to the aforementioned possibilities, differences in activity between atrop-abyssomicin C and abyssomicin C against Gram-positive bacteria and mycobacteria may be also due to differences in uptake, metabolic inactivation, or efflux of abyssomicins by different species of bacteria.

Abyssomicin I is an inactive new abyssomicin variant, lacking the Michael acceptor system and derived from a different methylation incorporation pattern within the polyketide biosynthesis pathway [37]. Chemical derivatization of abyssomicin by selective oxidation (Section 5.6) yields its oxidized derivative, and restores the Michael acceptor system along with antimicrobial activity (Figure 2, Table 1) [37]. Additionally, both abyssomicin I and its oxidized derivative inhibited the tumor cell invasion at non-cytotoxic concentrations [37]. This emphasizes the role of the Michael acceptor system for antimicrobial activity (Table 1) and cytotoxicity (Table 3); and the role of the methyl at C12 for tumor cell invasion properties not seen in other active abyssomicins (Figure 11).

Atrop-*o*-benzyl-desmethylabyssomicin C is a desmethyl benzyl ether of abyssomicin C, lacking three methyl structures (at C4, C6, and C13) (Figure 2) [42]. This synthetic analogue retained antimicrobial activity, while its cytotoxicity decreased by two to three-fold compared to atrop-abyssomicin C, both on HeLa and PBC cells (Table 3). This implies the involvement of those three methyl groups in toxicity (Figure 2 and Figure 11) [42]. The three methyl groups in the natural product are important for specific binding to small hydrophobic subsites of the putative target, implicated in its cytotoxic action [33]. Unfortunately, while demethylation at C4, C6, and C13 reduced cytotoxicity in HeLa and PBC cells, atrop-*o*-benzyl-desmethylabyssomicin C is still cytotoxic for healthy somatic cells at concentrations required for the antibacterial activity, especially since demethylation also diminished antimicrobial activity [42].

Additional chemical derivatization and SAR studies demonstrated that neither the free C11 hydroxy group nor the C3 carbonyl group is needed for antibiotic activity. It also showed that the replacement of the C11 hydroxy group by a benzyl ether (as in the benzyl ether derivative of atrop-abyssomicin C) enhances antimicrobial activity and cytotoxicity with respect to the natural (−)-atrop-abyssomicin C (Table 1) [34]. 

The high Michael acceptor potency of abyssomicins responsible for the biological activity suggests a low in vivo half-life. With the discovery of abyssomicin J and atrop-*o*-benzyl-desmethylabyssomicin C, this hurdle can be overcome. While atrop-*O*-benzyl-desmethylabyssomicin C is a less potent Michael acceptor, which may enhance a short half-life, its decreased antimicrobial effect implies that it is cytotoxic at concentrations required for antibacterial activity. On the other hand, effective doses may be less toxic with abyssomicin J, as it is a prodrug, characterized by a thioether function at C12 (Figure 2 and Figure 11). Abyssomicin J undergoes reverse Michael addition by means of in situ enzymatic oxidation via the P450 enzyme to yield atrop-abyssomicin C in vivo through a transformation including plausible sulfonic and sulfinic acid intermediates [35]. This thioether Michael adduct prodrug paradigm (Figure 10) offers a promising new approach to stabilize highly reactive Michael acceptors, thereby enhancing bioavailability, selectively delivering the antibiotic and improving therapeutic potential [24]. SAR requirements are summarized in Figure 11.

### 7.2. Viral Induction

Abyssomicin 2 is the most active among abyssomicin 2–5 exhibiting latent HIV reactivation activities [38]. Even though the Michael acceptor system is not present in abyssomicin 3–5, they are still viral inducers [38]. While the latent HIV potencies of abyssomicin 3 and 4 are marginal, they are not completely abolished. This not only emphasizes the importance of the Michael acceptor system for viral induction [38], but also points towards other SAR requirements that are yet to be elucidated. Abyssomicin 3–5 are devoid of the Michael acceptor system and would likely not exhibit antimicrobial activity. Abyssomicin 2 and the oxidized derivative of abyssomicin I are enantiomers with an intact Michael acceptor system, both exhibiting antimicrobial activity [37,51]. Since abyssomicin 2 activates latent HIV, the oxidized derivative of abyssomicin I may also have a similar activity. Moreover, other antimicrobially active abyssomicins may also be activators of latent HIV, although these hypotheses remain to be experimentally proven. 

Despite having latent HIV inducer activities, abyssomicin 2 exhibited anti-HIV activities in a different experiment [51]. Moreover, neoabyssomicins A–C were found to promote HIV-1 viral replication [51]. SAR trends relevant to cytotoxicity and antibacterial capabilities are, by now, well defined, and seem to be straightforward. Nonetheless, SAR trends for HIV-1 viral replication, anti-HIV, and latent HIV inducer activities remain to be deciphered. 

## 8. Summary and Outlook

The investigation of terrestrial and marine members of the order *Actinomycetales* led to the discovery of the novel abyssomicin family, which belongs to the class of tetronate antibiotics, as they are small Class I spirotetronate polyketides. At present, this family comprises thirty-two natural members (note S19, supplemental data) and numerous synthetic analogues. The interest in the abyssomicin pharmacophore was fueled by its antibacterial activity targeting mycobacteria and Gram-positive bacteria like *S. aureus*, including MRSA and VRSA. Notably, in addition to their antibacterial activities, this novel family also exhibits latent HIV reactivation and antitumor activities. The antimalarial activity of this class of compounds remains to be explored. Impressive synthetic, chemical, and biological efforts were combined to decipher the SAR requirements and the mechanism of action of abyssomicins at the molecular level. Today, total synthesis of abyssomicins (namely abyssomicin C and atrop-abyssomicin C) has been reported, along with semi-synthesis of different natural abyssomicins. In addition, advances in microbial biosynthesis and genetic engineering [56,74,75,76,77,78,79,80] are applicable to actinomycetes [56,78,79,80], and should offer a potential solution to large-scale production or semi-synthesis of abyssomicins. These genetic engineering approaches (including microbial genome sequencing mining, metagenomic studies, and combinatorial biosynthesis (e.g., of non-ribosomal peptide synthetase and PKSI multi-enzymes)), coupled with advances in bioinformatics and mass spectral analyses of secondary metabolites, can be employed to not only improve the product titers for isolation, characterization, commercial product development, and manufacturing of abyssomicin analogues, but also, to activate and enhance the expression of genes involved in the synthesis of abyssomicin, and to increase their antimicrobial activity [56,78,79,80].

The mechanism of action by which the abyssomicin family exerts antimicrobial and antimycobacterial activity consists of a hetero-Michael addition based ADCS enzyme-trapping mechanism, where the abyssomicin pharmacophore, being a substrate mimetic of chorismate, covalently traps the Cys263 of the PabB subunit of ADCS, which yields a sulfur-bound abyssomicin D-like structure in the process. Active abyssomicins are antagonists of chorismate, inhibiting irreversibly pABA synthesis within the chorismate pathway, and consequently inhibiting folate synthesis within the SCF metabolic pathway. Abyssomicins validate the chorismate pathway as a novel antimicrobial target within the SCF biosynthetic pathway, and shed light on the abyssomicin family as its first natural inhibitors. The abyssomicin class may be the novel structure sought to replace the iterative cycles of antibiotics in order to treat resistant pathogens.

An intact Michael acceptor moiety and an 11-membered cyclic structure are essential structural motifs for the observed antibacterial activity. However, the presence of such a reactive enone-moiety hampers the potential of abyssomicins as antimicrobial leads, since it can give rise to both cytotoxicity (via non-selective alkylation of cellular nucleophiles) and a short half-life. Indeed, active abyssomicin compounds are characterized by moderate MICs, a short half-life, and high cytotoxicity. The discovery of abyssomicin J may help enhance the short half-life of abyssomicins as it acts like a prodrug. The thioether function of abyssomicin J stabilizes the highly reactive Michael acceptor system, thereby enhancing bioavailability and improving therapeutic potential. 

Moreover, molecular tailoring and SAR studies helped better understand the motifs responsible for both the activity and toxicity of this class of compounds. For instance, the synthesis and biological evaluation of atrop-*o*-benzyl-desmethylabyssomicin C give hope that analoging programs may provide a new abyssomicin structure with an improved efficacy and lower toxicity. Considering atrop-*o*-benzyl-desmethylabyssomicin C and abyssomicn J in such analoging programs may ultimately yield a superior abyssomicin analogue that is clinically effective at non-cytotoxic concentration. Meanwhile, the abyssomicin pharmacophore remains an antimicrobial lead that is effective against mycobacteria and resistant bacteria, including MRSA and VRSA.

## Figures and Tables

**Table 1:** Minimum inhibitory concentrations (MICs) (μg/mL) of active abyssomicins and derivatives against different Gram-positive bacteria.

	MIC in μg/mL							
Compound	MRSA N315	MRSA 100b	MRSA 2775	VRSA Mu50	*M. luteus* ATCC 9343	*B. subtilis* PCI 219	*S. aureus* IFO 12732	MRSA ATCC 33591
Abyssomicin C	4 [30,31,32,33]	ND	ND	13 [27,28,29,53]	ND	ND	ND	5.2 [53]
Atrop-abyssomicin C	~5 [33]	20 [42]	20 [42]	ND	ND	ND	ND	3.5 [53]
(−)-Atrop-abyssomicin C	~5 [30,31,32,33]	20 [34]	20 [34]	ND	ND	ND	ND	ND
Benzyl ether derivative of atrop-abyssomicin C	ND	8 [34]	10 [34]	ND	ND	ND	ND	ND
Chloro derivative of atrop-abyssomicin C	ND	15 [58]	15 [58]	ND	ND	ND	ND	ND
First diastereoisomeric MOM ethers derivative of atrop-abyssomicin C	ND	12 [34]	15 [34]	ND	ND	ND	ND	ND
Second diastereoisomeric MOM ethers derivative of atrop-abyssomicin C	ND	12 [34]	15 [34]	ND	ND	ND	ND	ND
Atrop-*O*-benzyl-desmethyl abyssomicin C	ND	44 [42]	58 [42]	ND	ND	ND	ND	ND
Oxidized derivative of abyssomicin I	ND	ND	ND	ND	29 [37]	29 [37]	29 [37]	ND
Acetyl abyssomicin C	~8 [33]	ND	ND	ND	ND	ND	ND	ND
3-Dithiolane atrop-abyssomicin C	~32 [33]	ND	ND	ND	ND	ND	ND	ND
Dithiolane abyssomicin C	ND	ND	ND	ND	ND	ND	ND	17 [45]
	**MRSA 1862**	**MRSA 991**	**MRSA 669**	**MRSA A1**	***M. luteus* ML01**	***B. thuringiensis* BT01**	***S. aureus* ATCC 29213**	***E. faecalis* ATCC29212**
Abyssomicin 2	14.5 [51]	58 [51]	>230 [51]	115 [51]	3.6 [51]	7.2 [51]	14.5 [51]	14.5 [51]

ND Not determined.

**Table 2:** MICs (μg/mL) of active abyssomicins and derivatives against different mycobacteria strains.

	MIC (μg/mL)		
Compound	*M. Smegmatis* mc2155	*M. Bovis* BCG	*M. Tuberculosis* H37Rv
Abyssomicin C [35]	ND	~2	ND
(−)-Abyssomicin C [36]	~10	~2.5	~1
(+)-Abyssomicin C [36]	~38	~20	ND
(−)-Atrop-abyssomicin C [36]	~20	~5	~2.5
(+)-Atrop-abyssomicin C [36]	~38	~10	ND
Abyssomicin J [35]	ND	3.125	ND

ND Not determined.

**Table 3:** Cytotoxicities of selected abyssomicin analogues.

	HeLa		PBC	
Compound	IC50	IC90	IC50	IC90	IC50
Atrop-abyssomicin C [34]	31.8	68.3	7.48	23	ND
Benzyl ether derivative of atrop-abyssomicin C [34]	18.4	45.5	6.21	15.1	ND
Chloro derivative of atrop-abyssomicin C [34]	18.4	40.1	6.16	17.4	ND
First diastereoisomeric MOM ethers derivative of atrop-abyssomicin C [34]	18.4	50.7	5.07	28.1	ND
Second diastereoisomeric MOM ethers derivative of atrop-abyssomicin C [34]	10.7	80.5	5.01	13.5	ND
Atrop-*O*-benzyl-desmethylabyssomicin C [42]	119,450	>1,000,000	3170	12820	ND
Oxidized derivative of abyssomicin I [37]	ND	ND	ND	ND	210
abyssomicin I [37]	ND	ND	ND	ND	11,000

IC50 and IC90 expressed in nM. Cytotoxicities determined on HeLa and PBC cells by the MTT assay [34]. The cell type used to determine the cytotoxicity of abyssomicin I and its oxidized derivative was not specified [37]. ND Not determined.

## Supplementary Materials

The following are available online: Notes S1–19.
