# Supplementary material for: Review on Abyssomicins: Inhibitors of the Chorismate Pathway and Folate Biosynthesis
Source: Molecules. 2018 Jun 6;23(6):1371. doi: 10.3390/molecules23061371 (PMC6100094; doi:10.3390/molecules23061371)
Supplement: Supplementary file 1 [file molecules-23-01371-s001.pdf]

Review on Abyssomicins: Inhibitors of the Chorismate Pathway and Folate Biosynthesis

Carmen Sadaka [1,*], Edmund Ellsworth [2], Paul Robert Hansen [3], Richard Ewin [4], Peter Damborg [1] and Jeffrey L. Watts [4,*]

[1] Department of Veterinary and Animal Sciences, University of Copenhagen, Stigboejlen 41870, Frederiksberg C, Denmark; pedam@sund.ku.dk
[2] Department of Pharmacology and Toxicology, Michigan State University, 220 Trowbridge Road, East Lansing, 48824, MI, USA; ellswo59@msu.edu
[3] Department of Drug Design and Pharmacology, University of Copenhagen, Universitetsparken 2100, Copenhagen, Denmark; prh@sund.ku.dk
[4] Zoetis Global Therapeutics Research, 333 Portage Street, Kalamazoo, 49007, MI, USA; richard.ewin@zoetis.com
* Correspondence: carmen.sdk@hotmail.com (C.S.); jeffrey.l.watts@zoetis.com (J.L.W.)

Supplementary Materials:

Note S1. The folic acid pathway consists of seven steps that can be divided into three branches leading to the formation of the active folate derivative. The first branch is for the formation of pterin ring through an extensive rearrangement of GTP followed by dephosphorylation, aldolase, and kinase steps. The second branch is for the addition of para-aminobenzoic acid (pABA), followed by the addition of glutamate moiety (by dihydropteroate synthase (DHPS) and dihydrofolate synthase (DHFS), respectively) to form the backbone of folate derivative. The third and final step is for the formation of the active folate form through a reduction of 7,8-dihydrofolate to 5,6,7,8-tetrahydrofolate by dihydrofolate reductase (DHFR) (Figure 1) [9].
Note S2. The function of the shikimate pathway is to provide chorismate (Figure 1) [11,12]. Although the shikimate pathway branches out at many points, chorismate constitutes its only end product [8]. Chorismate is a precursor of pABA and other intermediate metabolites (prephenate, anthranilate, aminodeoxychorismate, isochorismate), as it enters five distinct pathways leading to their synthesis [8].
Note S3. The function of the chorismate pathway is the biosynthesis of pABA (Figure 1) [9].
Note S4. Sulfonamides competitively inhibit dihydropteroate synthase (DHPS) enzyme in the folic acid pathway by their structural analogy with the normal substrate pABA but can also function as alternative substrates for DHPS. This results in the formation of sulfa-containing pterin adducts, which cannot be further metabolized, thereby stopping folate synthesis, and ultimately, cell growth [3,10,23,26].
Note S5. Trimethoprim (TMP) is a synthetic diaminopyrimidine that competitively inhibits the dihydrofolate reductase (DHFR) enzyme in the folic acid pathway by its structural analogy to the pterin portion of its natural substrate, dihydrofolic acid. The competitive inhibition of DHFR by trimethoprim blocks the production of the biologically active form of tetrahydrofolic acid from dihydrofolic acid [3,10,23,26].
Note S6. The synthesis is described in detail in K.C. Nicolaou, S.T. Harrison and J.S. Chen 2008 and in K.C. Nicolaou and S.T. Harrison 2007 [12,26].
Note S7. Bihelovic and coworkers reported a formal synthesis of abyssomicin B, D, G, J, K, and L in their total synthesis of atrop–abyssomicin C, which also yielded a total synthesis of abyssomicin H. More details about the approach are available in section 5.2. Isolation and

synthesis of atrop-abyssomicin C of this manuscript. The complete details of the synthesis can be found in F. Bihelovic and R. N. Saicic (2012), and in F. Bihelovic and coworkers (2013) [54,58].

Note S8. Please refer to Section 5.1 ''Synthesis of abyssomicin B–D'' in this review for more details on the Sorensen and coworkers' and Nicolaou and coworkers' syntheses.

Note S9. During this reinvestigation, the abyssomicins were purified as described previously by Riedlinger and coworkers, [33] but using acid free solvents instead [30]. Nonetheless, atrop-abyssomicin C depleted during previous purifications or was converted to abyssomicin C by use of acidic HPLC solvents. In addition, the extremely high similarity of both compounds with regards to retention time and UV spectra obscured an earlier observation of this loss [32].

Note S10. The details of this synthesis can be found in F. Bihelovic and R. N. Saicic (2012) and in F. Bihelovic and coworkers (2013) [34,41].

Note S11. Wang and coworkers screened a library of marine-derived bacteria (4024) and fungi (533) for growth inhibitory activity against Bacille Calmette Guerin (BCG), an attenuated strain of the bovine tuberculosis *Bacillus M. bovis*. They detected 27 (0.6%) extracts with anti-BCG activity, including a South China Sea deep-sea (−2733 m) sediment-derived actinomycete, *Verrucosispora sp.* (MS100128). Bioassay directed fractionation of a large scale (21 L) culture of MS100128 yielded three new members of the rare class of abyssomicin polyketides, abyssomicins J, K, and L, and the four known abyssomicins B, C, D, and H [35].

Note S12. Exposure of abyssomicin C to 0.1 M Na_2S yielded three products in a near quantitative conversion: abyssomicin J (major product), a thiol intermediate 12 (minor product) and its oxidation product, a sulfonic acid intermediate 14 (minor product) [35].

Note S13. Exposure of abyssomicin C to 0.05 M NaOH yielded a single product, abyssomicin K [35].

Note S14. Exposure of abyssomicin C to 0.5 M TFA led to a mixture of abyssomicin K with a novel semi-synthetic isomer 15. Exposure of the new semi-synthetic analogue 15 to 0.05 M NaOH resulted in quantitative conversion into abyssomicin K [35].

Note S15. Exposure of abyssomicin C to 0.5 M TFA in MeOH yielded a single product, abyssomicin L. This reaction also proceeded at a far slower rate, without exposure to acid, during handling and storage of abyssomicin C in MeOH [35].

Note S16. Exposure of a $MeCN/H_2O$ solution of abyssomicin J to the oxidizing reagent oxone (as a chemical P450 surrogate) yielded four products: atrop-abyssomicin C and three analogues (a sulfoxide analogue, a sulfone analogue, and a sulfonic acid analogue). Abyssomicin C was not formed [35].

Note S17. The formation of tetronate is composed of the following steps: (i) transfer of a glycerol moiety from d-1,3-biphosphoglycerate to a discrete ACP (*abmA3*) as catalyzed by a glyceryl-*S*-ACP synthase (*abmA2*), leading to glyceryl-*S*-ACP; (ii) binding of the glyceryl-*S*-ACP to the nascent polyketide chain and detachment of the polyketide from the PKS, generating the linear hydroxymethyl tetronate ring as catalyzed by a ketoacyl-*S*-ACP synthase (*abmA1*); (iii) exomethylene installation via an acylation-elimination process accomplished by an acyltransferase E2 component of 2-oxoacid dehydrogenase multienzymes (*abmA4*) and an α/β hydrolase fold protein (*AbmA5*) [52].

Note S18. This observation was borne out in kinetics experiments studying the reduction of both compounds with an NADH analog [33].

Note S19. Including abyssomicin B–E, abyssomicin G–L, ent-homoabyssomicin A and B, atrop-abyssomicin C, abyssomicin 2–5, abyssomicin M–X, and neoabyssomicin A–C. Abyssomicin C, J and atrop-abyssomicin C, abyssomincin 2 are the only antimicrobially active natural abyssomicins.
